# Are the Symptoms of Parkinsonism Cortical in Origin?^☆^^☆☆^

**DOI:** 10.1016/j.csbj.2016.10.006

**Published:** 2016-10-27

**Authors:** Gordon W. Arbuthnott, Marianela Garcia-Munoz

**Affiliations:** OIST Graduate University, Brain Mechanisms for Behaviour Unit, Okinawa, Japan

**Keywords:** Parkinson's disease, Deep brain stimulation, Layer I, Motor cortex

## Abstract

We present three reasons to suspect that the major deleterious consequence of dopamine loss from the striatum is a cortical malfunction. We suggest that it is cortex, rather than striatum, that should be considered as the source of the debilitating symptoms of Parkinson's disease (PD) since:

1. Cortical synapses onto striatal dendritic spines are lost in PD.
2. All known treatments of the symptoms of PD disrupt beta oscillations. Oscillations that are also disrupted following antidromic activation of cortical neurons.
3. The final output of basal ganglia directly modulates thalamic connections to layer I of frontal cortical areas, regions intimately associated with motor behaviour.

These three reasons combined with evidence that the current summary diagram of the basal ganglia involvement in PD is imprecise at best, suggest that a re-orientation of the treatment strategies towards cortical, rather than striatal malfunction, is overdue.

## Highlights

• Suggested experimental contributions support the proposal of a cortical participation in PD.
• DBS produces antidromic activation of motor cortex and desynchronizes beta oscillations.
• Loss of dopamine decreases dendritic spines in the striatal D2 projection neurons.
• Motor thalamus distributes terminals into frontal cortex layer I.
• Thalamocortical-layer I activity increases with locomotion.

At least three generations of scientists have already devoted their life to understanding PD. There is no doubt that important achievements have been made during this time. The development of the animal models necessary to acquire new data and advances in the treatment of patients, are two of the many significant contributions. Here, we briefly review some accomplishments in the field to emphasize that the contribution of striatum and its output pathways, although significant, might be modest compared to cortical abnormalities evidenced by the pathological oscillatory activity. Further, the loss of synaptic contacts in the striatum likely arises from the corticostriatal pathway. Cortical participation is accentuated by its antidromic activation by the successful treatment of the disease after applying DBS. Lastly, the participation of the return pathway from the basal ganglia to the motor thalamus and its input to layer I found experimentally, suggests possible therapeutic procedures, although none are yet well developed in treatment strategies.

## 1 Approaches to the Treatment of PD

With the discovery of dopamine as the neurotransmitter lost in Parkinson's disease (PD) [30] it seemed obvious that the loss of dopamine innervation to striatum was the source of symptoms and the cause of the disease [2], [21]. Naturally, treatments initially centred on replacing the missing dopamine with L-Dopa. This pharmacological approach was successful to some extent, the progression of the disease was slowed and patients lived twice as long as before. Nonetheless, the quality of life did not improve as much as desired; for review see Paul and Borah [59]. Subsequently, the postulation that PD symptoms are a consequence of a fundamental imbalance between the two striatal output pathways [1], provided the basis for new neurosurgical therapy. Sporadic surgical procedures such as interruption of pyramidal tract, motor cortex or cerebral peduncle, were then replaced by the successful ablation of basal ganglia outputs as therapy for PD. A reassuring reversal of symptoms, at least for a period of 2 years, was observed on the side contralateral to surgery. Lesions were performed either in the ventroposterolateral globus pallidus or the ventral intermediate nucleus of the thalamus [47]. Other successful targets were the globus pallidus externus or subthalamic nucleus in both rodents and monkeys [10]. Lesions of the subthalamic nucleus were too risky for humans, since it is penetrated by the branch of the internal carotid artery that feeds most of the forebrain. Added to this surgical risk was the debilitating side effect of a lesion induced-hemiballism that only a few patients did not present, for review see Guridi and Obeso [38]. Pallidotomy and thalamotomy of ventrolateral or ventromedial nuclei became popular, although not without controversies about reproducible successful locations and the need of more controlled studies [48].

## 2 The Advent of Deep Brain Stimulation (DBS)

Instead of a permanent lesion, a modern strategy for the recovery of PD symptoms, became the high frequency stimulation of the subthalamus [8], [27]. This deep brain stimulation (DBS) technique produced spectacular relief of debilitating symptoms for many patients, and became an established treatment for advanced PD. The disease still progresses but the patients are in a much better situation for another few years, until cognitive symptoms and other complications emerge [55].

Years of basic science research performed in patients, or animal models, have yielded contradictory results about how the therapeutic effects of DBS are achieved. Logically, electrical stimulation had to affect excitable tissue, i.e., local neurons, presynaptic and postsynaptic neuronal processes or fibres of passage.

Initially DBS was supposed to produce a reversible functional neuronal silencing. However, the possibility of stimulation inducing ‘depolarization block’ [7], [8] seemed unlikely since ‘depolarization block’ in motorneurons just moves the site of stimulation a few nodes down the axon [29], [54]. DBS did not inhibit the production of action potentials [53] as reasonably proposed [25]. Instead, stimulation resulted in neurons producing action potentials that loosely followed different ranges of frequency of stimulation –entrainment [6], [20]. Entrainment is consistent with the capacity of neurons to fire much faster than 125 Hz, though admittedly for short periods.

## 3 DBS and Antidromic Cortical Stimulation

Over the past decade, another explanation emerged for the beneficial effects of DBS: stimulation of fibres of passage or axonal terminal fields. It was proposed that stimulation of cortical fibres under the electrode could be responsible for the therapeutic effect of DBS. Initial studies of neuronal excitability properties (e.g., chronaxie and refractory period) concluded that the large myelinated axons are excited before activation of cell bodies and low calibre axons, for review see [60]. Chronaxie and refractory period determined in patients undergoing thalamic DBS revealed a short chronaxie (50 μs) with short refractory periods [5], [66]. These values of the excitability properties allowed axons to follow the high frequencies (> 100 Hz) of subthalamic DBS. Subsequently, it was observed in patients, that single stimuli induced an antidromic positive slow electroencephalographic (EEG) wave with a constant short latency [5], [39]. In anaesthetized rats, it was possible for us to demonstrate that the slow EEG wave peaked in coincidence with antidromic activation of layer V cells in the motor cortex [51]. These results led to the hypothesis that DBS produces antidromic activation of motor cortex. Consistent with a cortical activation related to the positive effects of DBS, is the report that optogenetic activation of layer V neurons in frontal cortex is effective in reducing symptoms in 6-hydroxydopamine treated mice, whereas direct cell body optogenetic stimulation, or inhibition, in the subthalamic nucleus does not reverse the behavioural effects of dopamine loss [37]. Moreover, recently it was reported that only specific optical stimulation of axons descending from motor cortex layer V to subthalamic nucleus (hyperdirect pathway, see below) reduced behavioural signs of a unilateral dopamine depletion [62]. This is congruent with our findings that in freely moving rats, only stimulation that generated the EEG antidromic potential, was sufficient to recover movement in rats made akinetic by application of dopamine receptor antagonists [23]; for review see [17].

## 4 Abnormalities in Cortical Function Associated to PD

Cortical efferent connections to basal ganglia form three different pathways: pyramidal, intratelencephalic and hyperdirect [28], [63]. They mainly originate in layer V of the motor and premotor cortex in the primate [58]. Neurons from pyramidal and intratelencephalic pathways connect to striatal neurons and the hyperdirect pathway connects cortex to the subthalamic nucleus with the shortest latency. Alterations in corticostriatal connectivity have been repetitively mentioned in association to PD [11], [16]. Here we want to emphasize other somewhat different and not so familiar aspects that involve the output of basal ganglia to motor thalamus and then to cortex layer I, as another source of cortical dysfunction in PD.

### 4.1 Beta Oscillations

An important characteristic of PD is the significant increase in beta frequency oscillations. These oscillations (10 < 30 Hz) occur along cortex and basal ganglia in normal animals and humans, and are associated with cognitive as well as motor behaviour, for review see Stein and Bar-Gad [64]. We have shown that the cortical effects of stochastic antidromic action potentials is sufficient to desynchronise the dominant beta frequency in impaired animals and remove the coherence between the EEG and action potentials in cortical layer V [50]. Similarly, the PD model that involves selective degeneration of dopamine neurons by deletion of the mitochondrial protein PINK1, has recently being used to study the strength of correlation between motor cortical neurons. Carron et al. [18] reported an initial surge of coactive and synchronized cortical networks more than a year before the onset of motor symptoms. Consistently, this abnormal cortical network activity is reduced by antidromic activation of cortical neurons by subthalamic nucleus high frequency stimulation. The authors suggest that the ‘calming’ desynchronization might result from activation of collaterals of layer V neurons synapsing onto interneurons. Apparently, the stochastic activation of cortical output cells can be sufficient to account for the disruption of the beta frequency activity [50]. Kang and Lowery [45] modelling the action of antidromic driving of cortical networks, showed that frequency of 130 Hz is the most effective stimulation rate for antidromic spike production and disruption of beta oscillations. This result is interesting since 130 Hz tends to be the most effective frequency in patients [46] and 125 Hz in the rat animal model [50].

The interest in oscillatory activity associated to PD initiated in the early years of the century, for reviews see [64], [68] and now it seems to be culminating in the claim that ALL treatments for the symptoms reduce the increase in beta oscillatory activity [14], [22]. In a recent review, we propose that basal ganglia output to motor thalamus and then to layer I, can modify layer V activity and oscillatory cortical activity [35].

### 4.2 Cortical Layer V

The influence of the layer V cortical output to striatum is clearly established in PD. We would like to think that the loss of dendritic spines is an indication of cortical degeneration, but factors other than a cortical loss maybe operating. Nonetheless, it is likely that the cortical input, and not thalamic is drastically changed [72]. Antidromic activation of cortex produced by DBS may not be the only reason to consider that the cortical output is altered in PD. Already we had presented evidence that loss of striatal dopamine led to a decrease in dendritic spines likely produced by a loss of excitatory synapses to medium spiny neurons [41], [43]. By 1998, our quantitative stereological results confirmed that dopamine depletion induced the dendritic loss of spines, on which the excitatory terminals synapsed [42]. Conditions of post-mortem tissue occasionally impair adequate acquisition of tissue slices for electron microscopy to verify all findings from animal models, but in those cases for which post-mortem data is available it is clear that spines are also missing [65], [70]. Subsequently, and unexpectedly, we observed that the major loss of spines was in the striatal projection neurons that express dopamine D2 receptors. In the absence of dopamine, striatal output neurons were expected to have an increased, instead of a decreased, glutamatergic drive [3], [15], [19], [33], [56], [57]. Several possible explanations are available for the presence of increased activity in dopamine-D2 neurons even when glutamatergic synapses on spines and the spines themselves are missing. For instance, a— the glutamatergic drive remains active on those neurons even with the loss of synaptic sites in the dendritic spines [24], [34] b— the size of some remaining synapses onto D2 cells is enlarged (unpublished results), c— spillover of glutamate from distant terminals reaches extrasynaptic A_2A_/D2 receptors located in the plasma membrane [32].

The important anatomical and functional changes in dendritic spines in D2 striatal projection neurons underlines their importance, however, it must be also emphasized that striatal D1 neurons are also significantly altered. They play a role in the expression of sensitization to prolonged administration of L-DOPA, in the dopamine denervated animals that results in choreic, dystonic and ballistic movements called L-dopa-induced dyskinesia [31], [67].

### 4.3 Cortical Layer I

Layer I is significantly altered in PD, for instance, Gaspar et al. [36] reported a loss in staining for the dopamine marker tyrosine hydroxylase close to 70% in PD patients. When we traced the neuronal system on which we expected the major action of dopamine, the final pathway led back to the cortex; it was a disappointing conclusion of years of work: the ‘output’ returned within a few 100 μm of the origin in layer V [4]. The final basal ganglia output projects to motor thalamus (ventromedial nucleus —VM— in rodents). Tracing the terminal distribution of those VM neurons in rats, led to layer I of the frontal cortex [4]. A quarter of a century ago, when we were performing those anatomical experiments, physiological methods to examine those inputs were not available. As we previously discussed [35] now inputs to layer I are becoming accessible. Moreover, we have observed that layer I thalamocortical activity becomes synchronized and increases during locomotion and administration of haloperidol [44] as also seen in PD patients [40], [61], [69]. We argue that VM stimulation activates inhibitory layer I interneurons that can directly contact apical dendritic tufts of layer V pyramidal cells. This inhibitory influence on pyramidal neurons is counter to the main idea that the thalamic output from the basal ganglia is excitatory in cortex. However, considering that decreased inhibition has been reported in motor cortex of animal models of the disease [12], [13] and in PD patients [49] restoration of inhibition could improve proper execution of motor patterns.

The motor thalamus-layer I anatomical arrangement could underlie the complex changes in cortical rhythms characteristic of distorted output from the basal ganglia and be a source of the symptoms in PD [35]. Since the long terminal branches in layer I can cover several millimetres, a single axon could influence large areas in the rat cortex [4]. Activation of the thalamic input to layer I over motor cortex, probably leads to the successful extradural motor cortex stimulation recently used in some patients as an alternative to DBS [9].

## 5 Optimistic Perspective

Lindenbach and Bishop [52] reviewed the influence of cortex on PD and concluded that there are good clinical reasons to consider the importance of cortex in the generation of symptoms and of effective cortical stimulation as therapeutic in PD. Further experimental data is needed, but the success of transcranial magnetic stimulation and repetitive transcranial magnetic stimulation in relieving symptoms, admittedly only short term and susceptible to placebo effect, is encouraging [71]. Similarly, and perhaps more consistent with our thesis, was the evidence of recovery in monkeys made parkinsonian with MPTP injection after stimulation via an electrode over the dura in the motor sulcus [26] and reassuring, are the efforts to stimulate motor cortex in patients with extradural electrodes [9].

## 6 A Touch of Reality?

Moving the problem from striatum to cortex does not make it any easier! There are many fundamental questions about cortical networks that we still need to understand. It could be that the only way to influence the cortical networks effectively, is via antidromic driving from subthalamus. Some obvious research questions include:

How do thalamocortical axons in layer 1 influence layer V neurons?

Is the beneficial effect of extradural stimulation a direct consequence of layer 1 activation or are deeper layers involved?

Is the effect of extradural stimulation via layer 1 or through actions in deeper layers?

Are particular subgroups of interneurons associated with beta oscillations?

Can particular subgroups of interneurons be influenced pharmacologically to produce a beneficial effect?

Finally, it is obvious that modification of the already damaged brain may only alleviate the worst symptoms. The most important contribution will be to find a cure. To achieve that we first need to understand, and actively influence, the cause or causes of dopamine cell death.
